# Proteomic and deep sequencing analysis of extracellular vesicles isolated from adult male and female *Schistosoma japonicum*

**DOI:** 10.1371/journal.pntd.0008618

**Published:** 2020-09-28

**Authors:** Pengfei Du, Bikash R. Giri, Juntao Liu, Tianqi Xia, Christoph G. Grevelding, Guofeng Cheng

**Affiliations:** 1 Shanghai Veterinary Research Institute, Chinese Academy of Agricultural Sciences, Key Laboratory of Animal Parasitology, Ministry of Agriculture, China; 2 Institute for Parasitology, BFS, Justus Liebig University, Giessen, Germany; 3 Shanghai Tenth People's Hospital, Institute for Infectious Diseases and Vaccine Development, Tongji University School of Medicine, Shanghai, China; University of Würzburg, GERMANY

## Abstract

Schistosomes are the causative agent of schistosomiasis, which affects more than 200 million people worldwide. Unlike other trematode parasites, schistosomes (along with the Didymozoidae) have evolved separate sexes. Pairing of males and females is a prerequisite for female sexual development and subsequent egg production. However, the mechanisms underlying these processes remain poorly understood. Extracellular vesicles (EVs) have been shown to play important roles in many biological processes. In the present study, we characterized EVs isolated from adult male and female *Schistosoma japonicum*. Proteomic analyses of the isolated EVs revealed that some proteins are significantly enriched in male or female EVs. RNA-sequencing analysis of a small RNA population associated with EVs identified 18 miRNAs enriched in male and female *S*. *japonicum* EVs. Among these, *miR-750* was specifically enriched in female EVs. Additionally, the inhibition of *miR-750* by a miRNA inhibitor led to decreased egg production in female schistosomes cultured *in vitro*. Collectively, our results suggest that *miR-750* within female EV cargo may be involved in regulating ovary development and egg production in *S*. *japonicum* females.

## Author summary

Schistosomiasis is a neglected tropical disease caused by the genus *Schistosoma* and affects more than 200 million people worldwide. Previously, we and other groups found that *Schistosoma japonicum* can secrete extracellular vesicles (EVs) that are taken up by mammalian cells. Here, we characterized EVs isolated from adult male and female *S*. *japonicum* and found that some proteins and microRNAs (miRNAs) were significantly enriched in male or female EVs. More importantly, the inhibition of *miR-750*, which is specifically enriched in female EVs, resulted in decreased egg production. Overall, our study suggests that female EV miRNA cargo may play important roles in regulating female ovary development and egg production during male-female pairing in *S*. *japonicum*.

## Introduction

Schistosomiasis is mainly caused by the parasitic trematode worms *Schistosoma japonicum*, *Schistosoma mansoni*, and *Schistosoma haematobium*, and affects more than 200 million people in approximately 76 tropical and subtropical countries [1]. Currently, praziquantel is the only drug used to control schistosomiasis; however, the frequent and continuous use of this drug could result in resistance [2, 3]. Therefore, further studies are needed to identify potential drug targets and develop novel therapeutic strategies against schistosomiasis.

Unlike other trematode parasites, schistosomes have evolved separate sexes during evolution. In this context, male-female pairing is prerequisite for female sexual maturation and subsequent egg production. The large number of eggs produced by mature females plays a central role in the pathology of infection with this parasite and in disease spread. Previous research suggested that the sexual development of the female schistosome depends on appropriate signals provided directly or indirectly by the male during pairing [4–13]. Thus, understanding the mechanism of female development as a consequence of pairing may lead to novel concepts for impeding egg production to lessen the spread of schistosomiasis.

Extracellular vesicles (EVs) are small membrane-bound secreted vesicles, that are important regulators of many biological processes such as proliferation, apoptosis, migration, and immunity [14]. Some protozoan parasites such as *Trichomonas vaginalis* [15], *Trichomonas cruzi* [16, 17], and *Leishmania spp*. [18] have been shown to secrete exosomes into living hosts; these exosomes then regulate host cell functions. For example, *Leishmania* exosomes can selectively induce the secretion of interleukin-8 in host macrophages [19]. In helminths, Marcilla and co-workers demonstrated that two trematodes, *Echinostoma caproni* and *Fasciola hepatica*, can secrete exosome-like vesicles that can be taken up by host cells, suggesting their potential role in communication between the parasites and host [20]. Additionally, Cwiklinski et al. found that miRNAs are in *F*. *hepatica* EVs [21] and that these EVs are associated with immune regulatory function [22]. Our previous studies indicated that adult schistosomes secrete exosome-like vesicles that can be taken up by host peripheral blood immune cells; the miRNA cargos of these vesicles then regulate host gene expression [23]. Similarly, previous studies demonstrated that *S*. *mansoni* can secrete EVs enriched in small non-coding RNAs and proteins, some of these proteins are considered as vaccine candidates [24, 25]. In nematodes, Buck and coworkers demonstrated that *Heligmosomoides polygyrus* secretes exosomes that are internalized by host cells to suppress the immunological response *in vivo* [26]. Moreover, accumulated evidence indicates that EV miRNAs play key roles in many biological processes in cancer, including tumorigenesis, metastasis and drug resistance [22–24]. Overall, these studies suggest that EVs and their miRNA cargo play multiple roles in many biological processes.

To evaluate the roles of EVs and their miRNA cargo in regulating schistosome development and egg production, we isolated EVs from adult male and female *S*. *japonicum*, and characterized the isolated EVs by proteomic techniques and deep sequencing. We found that *miR-750* was specifically enriched in female EVs. Additionally, the inhibition of *miR-750* by miRNA inhibitor led to decreased egg production in female schistosomes cultured *in vitro*. Our results indicate that male or female EVs and their miRNA cargo from *S*. *japonicum* play important roles in regulating ovary development and egg production in females.

## Results

### Size distribution of EVs isolated from adult male and female *S*. *japonicum*

**Fig 1:**
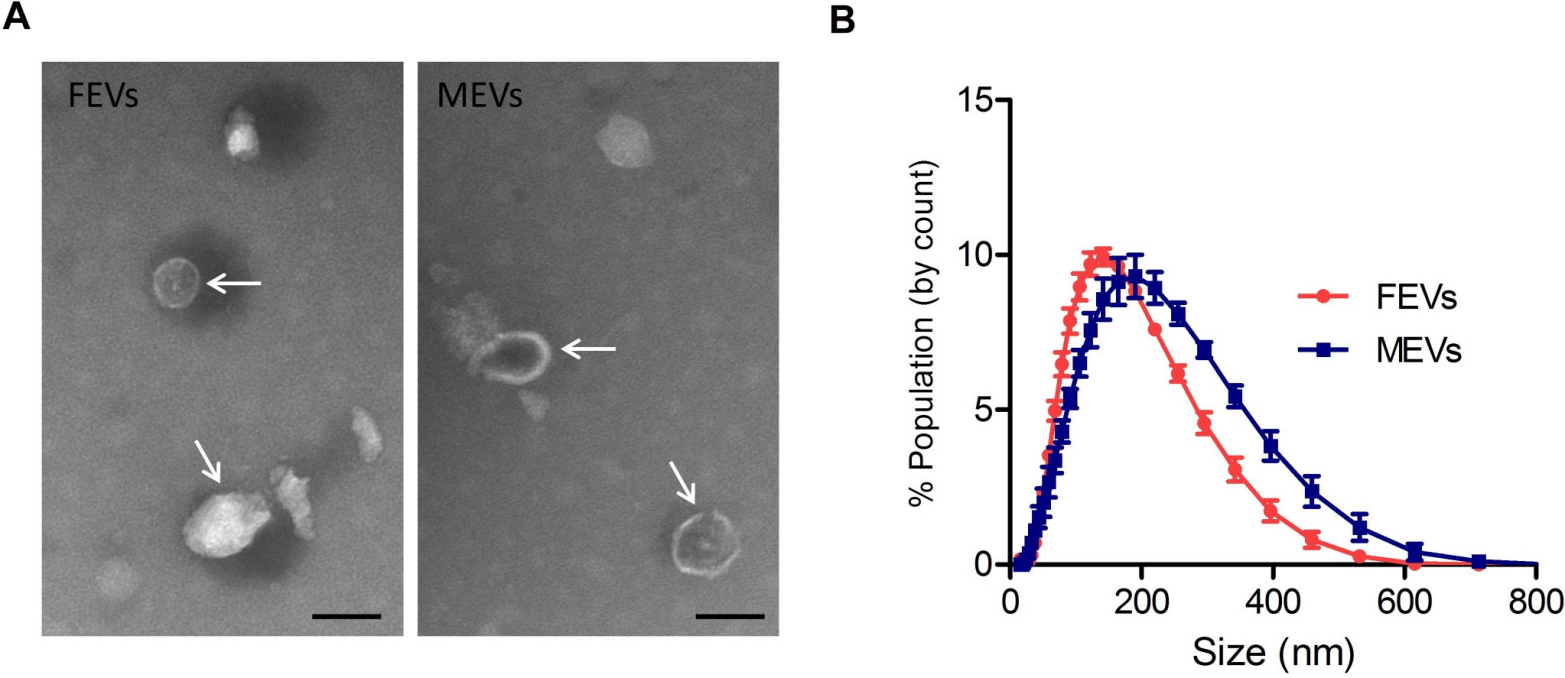
Morphology and size of extracellular vesicles (EVs) isolated from adult male and female *S*. *japonicum*. A) Morphological characterization of SjEVs determined by transmission electron microscopy. Arrows indicate isolated EVs, bars indicate 200 nM. B) Representative results of the size distribution of isolated EVs determined using a Zetasizer Nano ZS from three independent isolate of each gender. MEVs, EVs isolated from males; FEVs, EVs isolated from females.

Using a previously developed protocol for schistosome EV isolation [27, 28], we isolated EVs from adult male and female *S*. *japonicum* (SjEVs) collected from rabbits infected with *S*. *japonicum* cercariae at 28 days post infection. Transmission electron microscopy (TEM) was preformed to observe the morphology of the isolated SjEVs (Fig 1A). Particle size measurements indicated that the isolated SjEVs ranged from 100 to 400 nm in size (Fig 1B), which is consistent with our previous observation [23].

### Proteomic analysis of proteins associated with adult male and female EVs

**Fig 2:**
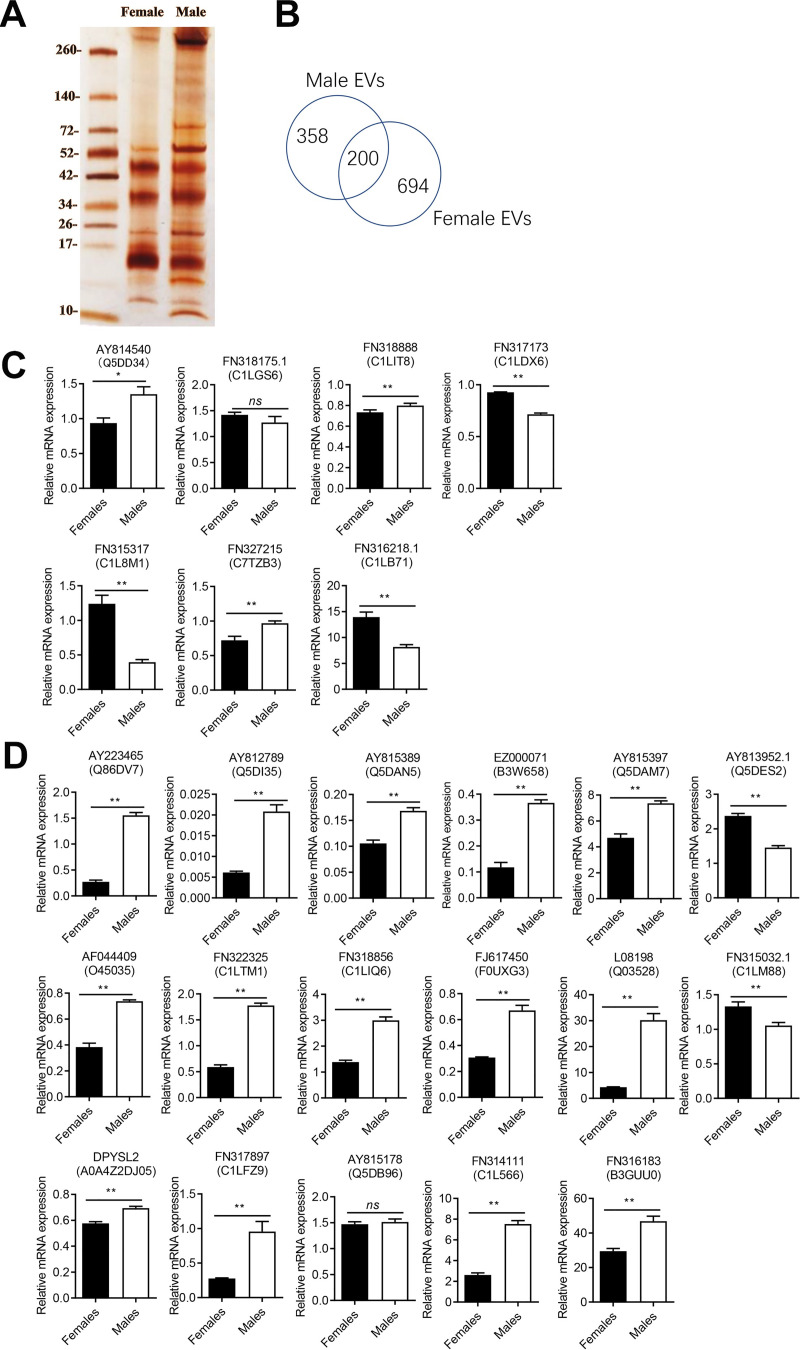
Characterization of proteins associated with female or male extracellular vesicles from *Schistosoma japonicum*. A) SDS-PAGE analyses of isolated male and female *S*. *japonicum* (SjEVs) by silver-staining. EVs isolated from male (1.5 μg) and female *S*. *japonicum* were separated by 4–20% SDS-PAGE gel and then stained with sliver. B) Venn diagram of identified proteins from male and female EVs. C) Reverse transcription–quantitative polymerase chain reaction (RT-qPCR) analysis of the transcript levels of selected proteins specifically detected in female EVs of *S*. *japonicum*. D) Reverse transcription–quantitative polymerase chain reaction (RT-qPCR) analysis of the transcript levels of selected proteins detected between male and female EVs of *S*. *japonicum*. For panel C and D, the data illustrate representative results and show the mean and standard errors of an experiment carried out in triplicate. * *P* ≤ 0.05 and ** *P* ≤ 0.01.

The proteins isolated from male and female SjEVs were separated by sodium dodecyl sulfate-polyacrylamide gel electrophoresis (SDS-PAGE) and then visualized by silver staining (Fig 2A). The protein components of the *S*. *japonicum* EVs were determined by shotgun liquid chromatography-tandem mass spectrometry (LC-MS/MS) analyses. The MS data search results identified a total of 558 proteins from male SjEVs (S1 Table) and 894 proteins from female EVs (S2 Table). Two hundred proteins overlapped among those identified in male and female SjEVs (Fig 2B, S3 Table). The EV marker proteins, such as Actin, Tubulin, HSP70, HSP90AA1, GAPDH, CD63, Annexin, Enolase, Aldolase, Lactate dehydrogenase, and Phosphoglycerate kinase were identified (S4 Table). Gene Ontology (GO) analysis indicated that the identified proteins are putatively involved in cellular process, metabolic process, biological regulation, developmental process and others.

Due to the lack of antibodies against specific proteins identified in male and female SjEVs, we performed quantitative polymerase chain reaction (qPCR) to determine the mRNA transcript levels of some of the identified proteins between male and female schistosomes. We selected 7 proteins specifically in female EVs (Fig 2C) and 17 proteins co-detected in male and female EVs (Fig 2D) for qPCR analysis. The results indicated that genes encoding all 24 proteins were able to be detected both females and males at the transcript levels. Among them, the mRNA transcript levels of 16 proteins were significantly increased in male schistosomes, whereas 5 mRNA transcripts were significantly increased in female schistosomes including ubiquitin specific peptidase (FN317173), saposin-like (FN315317), proteasome (AY813952.1), 20S proteasome subunit alpha (FN315032.1), and eukaryotic translation initiation factor 5A (FN316218.1) (Fig 2C and 2D). We noted that several proteins that specifically detected in female EVs (AY814540, FN318888 and FN327215) were shown to be highly expressed in males at transcript levels (Fig 2C).

### Identification of small RNA population associated with male and female SjEVs and validation of abundance of their miRNA cargo

**Fig 3:**
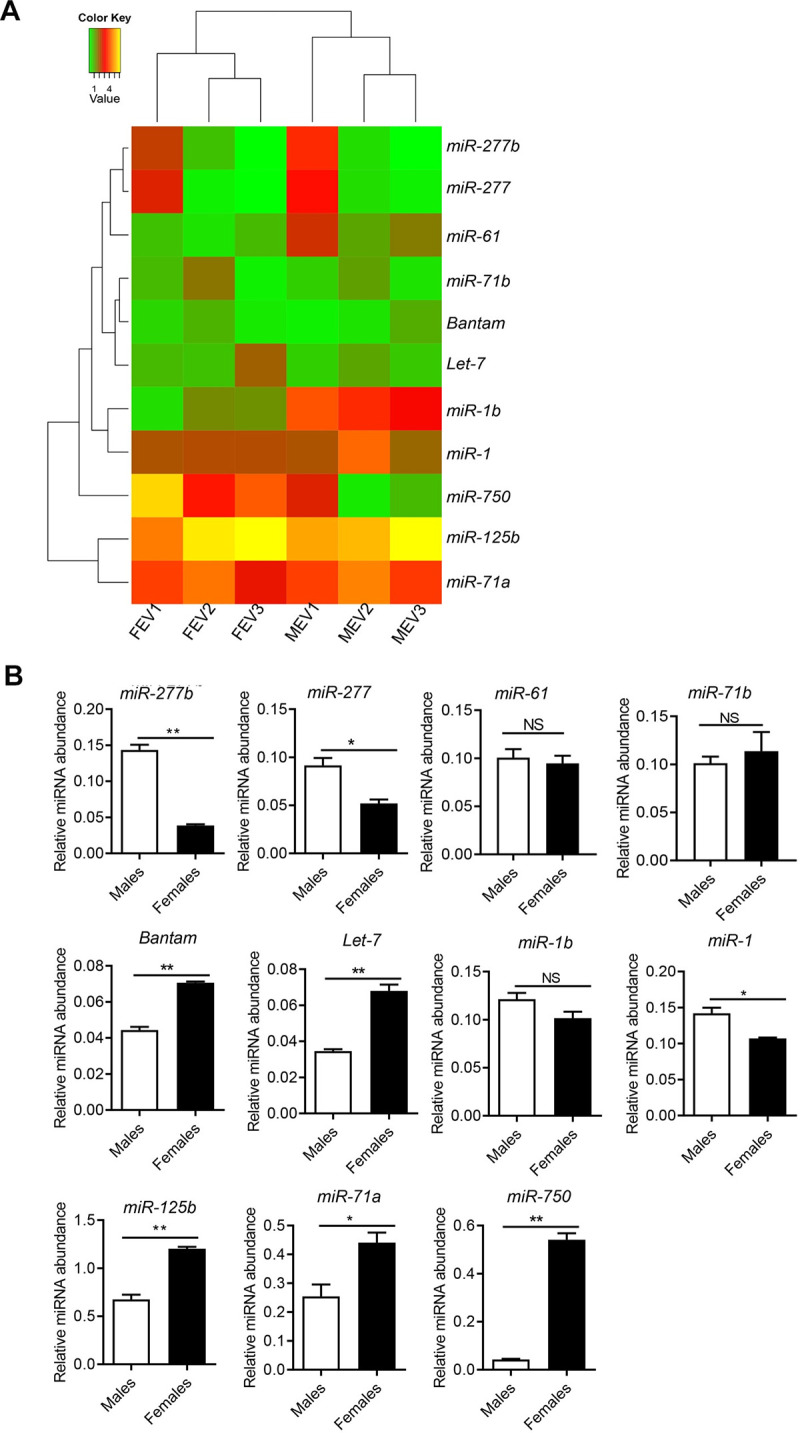
Identification of miRNAs associated with male and female extracellular vesicles (EVs) from *Schistosoma japonicum*. A) Heat map of miRNAs associated with females and males EVs of *S*. *japonicum*. Data illustrate the reads of SjEV miRNAs with reads ≥ 500 (at least two biological replications) for three biological replications (MEV1, MEV2, and MEV3 for males; FEV1, FEV2, and FEV3 for females). B) Validation of the abundance of miRNAs associated with male and female EVs from *S*. *japonicum* by RT-qPCR. Data illustrate representative results and show the mean and standard errors of triplicate analysis. * *P* ≤ 0.05 and ** *P* ≤ 0.01.

Small RNAs incorporated into EVs may play a regulatory role in exosome-mediated cell-cell communication. Therefore, we analyzed small RNA populations associated with male and female SjEVs by Solexa deep sequencing. The length distribution of the male or female EVs associated with small RNAs ranged from 20 to 24 nucleotides (nt). We noted that tRNA/rRNAs (average percentage of 57% for male SjEVs or female SjEVs), repeat-associated small RNAs (average percentages of 7% and 4% for male and female SjEVs, respectively), and miRNAs (average percentages of 5% and 10% for male and female SjEVs, respectively) were the dominant class of small RNAs. In the three biological replicates, 11 miRNAs with reads ≥ 500 in at least two biological replicates were shown in Fig 3A. The full list of identified miRNAs was provided in S5 Table. RT-qPCR was performed to validate the abundance of these 11 identified miRNAs in independently prepared male and female SjEVs. In total, 11 miRNAs were successfully amplified and the results indicated that *miR-277b*, *miR-277*, *bantam*, *Let-7*, *miR-1*, *miR-71a*, *miR-125b*, and *miR-750* differed in abundance between male and female SjEVs (Fig 3B) and the expression data from RT-qPCR and deep sequencing in general correlated well.

### Inhibition of *miR-750* led to decreased egg production in female schistosomes

**Fig 4:**
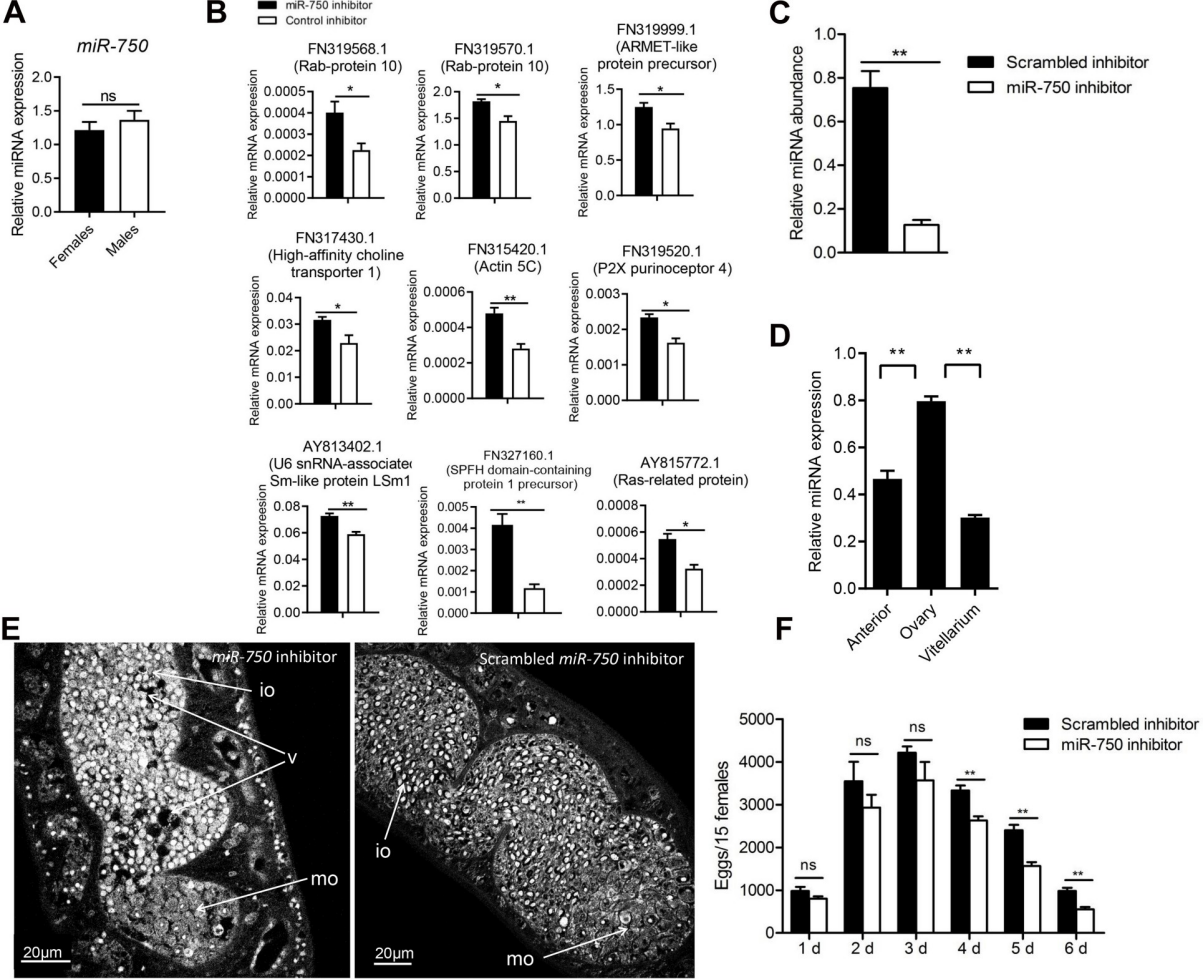
Inhibition of *miR-750* led to decreased egg production in female schistosomes. A) RT-qPCR analysis of *miR-750* expression in female and male schistosomes. ns means no statistically significant difference. B) RT-qPCR analysis of selected targets of *miR-750* in female schistosomes treated with *miR-750* inhibitor (antisense *miR-750*). Data show representative results and mean and standard errors from triplicate analysis. * *P* ≤ 0.05 and ** *P* ≤ 0.01. C) *miR-750* was significantly decreased in female schistosomes treated with *miR-750* inhibitor (antisense miR-750). Female schistosomes were electroporated either with *miR-750* inhibitor (antisense *miR-750*) or scrambled miRNA inhibitor and then cultured *in vitro* for 4 days. The total RNAs were isolated from treated worms for RT-qPCR analysis. Data show representative results and mean and standard errors from triplicate analysis. ** *P* ≤ 0.01. D) RT-qPCR analysis of the abundance of *miR-750* in three parts of dissected female schistosomes. Data show representative results and mean and standard errors from triplicate analysis. ** *P* ≤ 0.01. E) Suppression of *miR-750* resulted in morphological defects in female schistosomes treated with antisense *miR-750*. Female schistosomes were electroporated either with *miR-750* inhibitor (antisense *miR-750*) or scrambled miRNA inhibitor and then incubated for 4 days. *Schistosoma japonicum* worms were stained with carmine red and whole-mount preparations of worms were imaged by confocal microscopy. Io, immature oocyte; mo, mature oocyte; v, vacuoles. F) Inhibition of *miR-750* led to decreased egg production. Female schistosomes (10 worms) were electroporated either with miRNA inhibitor for silencing *miR-750* (3 μg) or scrambled *miR-750* inhibitor (3 μg) and then cultured in a well of 12-well cell culture cluster for 6 days. The numbers of eggs in each well were counted at 1, 2, 3, 4, and 5 days after electroporation by microscopy. Data illustrate representative results and show the mean and standard errors of triplicate wells.

In our previous study, we characterized miRNAs that were differently expressed between male and female *S*. *japonicum* and found that 14 and 4 miRNAs were enriched in male worms and female worms, respectively [29]. A comparison with our dataset revealed that highly expressed miRNAs in a specific gender were significantly enriched in the corresponding EVs. Interestingly, *miR-750* was specifically enriched in female EVs but it showed no significant difference between adult male and female *S*. *japonicum* (Fig 4A).

To examine any role of *miR-750* in regulating egg production in female schistosomes, we first predicted *in silico* targets of *miR-750* resulting in 375 targets and 617 targets by Targetscan and miRanda, respectively. There are 375 targets co-predicted from these two algorithms (S7 Table). GO analysis indicated that these co-predicted targets are putatively involved in cellular process, metabolic process, localization, biological regulation, and biological process. Kyoto Encyclopedia of Genes and Genome (KEGG) analysis indicated the putative targets are mainly involved in thiamine metabolism, purine metabolism, and oxidative phosphorylation (S8 Table). Next, we delivered a *miR-750* inhibitor (antisense *miR-750*) into *in vitro*-cultured adult female schistosomes by electroporation. Quantitative reverse transcription polymerase chain reaction (RT-qPCR) analysis indicated that the abundance of *miR-750* and some putative targets was significantly decreased in the treated worms (Fig 4B and 4C). We further dissected females into three parts (anterior, ovary, and vitellarium) and used RT-qPCR to determine the expression of *miR-750* in each part. The results indicated that *miR-750* was predominantly expressed in the region of ovary (Fig 4D). Notably, we observed remarkable morphological defects in the ovarian architecture, in most of the treated females including numerous vacuoles and damaged oocytes; in contrast, no alterations were found in the ovaries of females treated with the scrambled *miR-750* inhibitor (Figs 4E, S1 and S2). Monitoring of the time-course of egg production showed that inhibition of *miR-750* significantly reduced egg production *in vitro* over relatively longer culture periods (4, 5, and 6 days) (Fig 4F).

## Discussion

Previous studies demonstrated that the pairing with male schistosomes is necessary for the successful development of female schistosomes [30, 31]. Genomic, transcriptomic, and proteomic studies have identified differences in genes/proteins between male and female schistosomes, revealing the molecular effect of male-female paring on female development and egg production [7, 8, 32–34]. However, the mechanism underlying female development and maturation during male-female pairing remains unclear. EVs are crucial mediators of intercellular communication in mammals, invertebrates and even prokaryotes for regulating many biological processes [35]. In particular, small RNAs within EVs can regulate certain genes in recipient cells and organs in the same manner as hormones [23]. To evaluate the roles of SjEVs in regulating female development, we isolated SjEVs from adult male and female *S*. *japonicum* and then characterized the proteins and small RNAs associated with male and female SjEVs. Among others, we found that *miR-750* is enriched in female EVs; further studies suggested that *miR-750* is involved in the regulation of egg production in female schistosomes. Overall, our results indicate that miRNA cargo in female EVs may contribute to the regulation of ovary development and egg production in females.

In the present study, adult *S*. *japonicum* were collected from rabbits infected with *S*. *japonicum* cercariae and paired males and females were separated manually *in vitro*. To minimize the physiological stress caused by the *in vitro* culture and the environment, we collected conditioned medium after 2 h for EV isolation. MS analysis of the protein components of male and female EVs indicated that female EVs contain relatively more proteins than those of males, suggesting an association with different protein populations and/or components between male and female EVs. Due to lack of specific antibodies for schistosome proteins, we used qPCR to determine the transcript levels of some of these proteins. We found that the transcript levels of several proteins that specifically identified in female EVs showed significantly high expression in males including SJCHGC04312 protein (AY814540), Ubiquitin carboxyl-terminal hydrolase (FN318888) and KH-type splicing regulatory protein (FN327215), suggesting these proteins may be selectively loaded into female EVs. Ubiquitin carboxyl-terminal hydrolase 1 (UCH1) usually catalyzes the hydrolysis of COOH-terminal ubiquityl esters and amides. Recent study indicated that adriamycin-resistant MCF-7 cells can secrete exosomes containing ubiquitin carboxyl terminal hydrolase 1 and P-glycoprotein into the extracellular microenvironment to regulate cell functions[36]. In *S*. *mansoni*, it has been shown that UCH1 has a differential expression profile for different stages [37], suggesting its potential roles in the regulation of schistosome development. The KH-type splicing regulatory protein is an RNA-binding protein that recognizes the AU-rich elements (AREs) within the 3′UTRs of mRNAs and controls their stabilities in the cytoplasm[38] while its functions in *Schistosoma* remain unknown. These specifically enriched EV proteins associated with gender likely play important roles in regulating certain biological processes. Further characterization of their functions in schistosomes may reveal their potential roles in male-female pairing and egg production.

miRNAs act as important regulators within the EV cargo. In total, we noted 18 miRNAs with reads ≥ 500 in at least two biological replicates from male and female EVs. Compared to our previous results of miRNA expression between male and female *S*. *japonicum*, miRNAs highly expressed in a gender of schistosomes were also shown to be abundant in EVs isolated from the corresponding gender. Interestingly, *miR-750* was abundant in EVs isolated from female schistosome but did not significantly differ between male and female schistosomes. Consequently, *miR-750* is likely enriched in female EVs. The delivery of a *miR-750* inhibitor into *in vitro* cultured schistosomes by electroporation resulted in reduced egg production, particularly after relatively longer culture periods. These results suggest that *miR-750* could regulate egg production in female schistosomes. Ovary development is a consecutive process involving the activation or inhibition of many genes. A report suggested that cyclin B is essential for the maturation of ovaries and plays a critical role during oogenesis and spermatogenesis in mud crab (*Penaeus monodon*) [39]; moreover, it can be targeted by *miR-750*, which shows differential expression in undeveloped and developed ovarian tissues [40]. Dynamic transcriptomes of *S*. *japonicum* showed that female maturation involves insect-like hormonal regulation [7]. An ortholog of the allatostatin-A receptor, which is well known in insects, was identified in *S*. *japonicum*. In insects, its ligand allatostatin can inhibits the generation of JH, an acyclic sesquiterpenoid that controls insect development and reproduction by interacting with the molting hormone ecdysone [41]. The putative JH receptor ultraspiracle (USP) is a likely target of *miR-750* in *Apis mellifera* [42]; this indicates that *miR-750* is involved in hormone signaling, immunity and the stress response via regulation of the vitellogenin (Vg) gene. However, because of the lack of suitable techniques for genetically manipulating schistosomes, especially knockdown of miRNA expression, it is difficult to determine whether the enriched *miR-750* in female EVs is directly related to egg production in female schistosomes, or whether the effect is indirect. CRISPR/Cas9 technology, which has recently been successfully applied in *S*. *mansoni*, may be useful for genetically knocking-out key molecules involved in EV biogenesis and/or key miRNA cargo in *S*. *japonicum* [43].

In conclusion, we characterized EVs isolated from adult male and female *S*. *japonicum* by small RNAomics and proteomics analysis and found that *miR-750* was specifically enriched in female SjEVs. Further functional analysis suggested that *miR-750* contributes to female ovary development and egg production. Overall, our results support the fundamental role of EVs and their cargo, which includes regulatory RNAs, in schistosome reproductive biology.

## Materials and methods

### Ethics statement

All experiments involving mice and rabbits were carried in strict accordance with the recommendations in the Guide for the Care and Use of Laboratory Animals of the Ministry of Science and Technology of the People’s Republic of China. All animal procedures were approved by the Institutional Animal Care and Use Committee (IACUC) of the Shanghai Veterinary Research Institute, Chinese Academy of Agricultural Sciences and by the Animal Management Committee and the Animal Care and Use Committee of Shanghai Science and Technology Commission of Shanghai municipal government for the Shanghai Veterinary Research Institute, Chinese Academy of Agricultural Sciences, China (Permit number: SYXK 2016–0010).

### Animals, parasites and EV isolation

The life cycle of *S*. *japonicum* (Anhui isolate) was maintained using New Zealand rabbits and BALB/c mice and the intermediate snail host *Oncomelania hupensis* obtained from Center of National Institute of Parasitic Disease of Chinese Center for Disease Control and Prevention (Shanghai, China). Mice were challenged with 80–150 *S*. *japonicum* cercariae via abdominal skin penetration. New Zealand rabbits were percutaneously infected with approximately 1,500 *S*. *japonicum* cercariae (Anhui isolate, China). Schistosomes were collected from rabbits infected with *S*. *japonicum* cercariae at 28 days of post infection. Parasites were thoroughly and gently washed three times with 50 mL PBS (pH 7.4) and then male and female worms were separated manually. Next, male or female schistosomes were maintained in preheated RPMI-1640 culture medium (HyClone, Logan, UT) at 37 °C under 5% CO_2_ at a density of ~5 worm pairs /mL for 2 h. Following 2 h incubation, worms were microscopically examined to ensure that their teguments were intact. Parasites and pellets were removed by centrifugation at 2,000 × g and 14,000 × g for 30 min each at 4 °C, respectively. The culture medium was collected for EV isolation as described previously [28]. The isolated SjEVs were analyzed by transmission electron microscopy (TEM) as described previously [27], and their sizes were determined using a Zetasizer Nano (Malvern Instruments, Malvern, UK) [23].

### Analysis of isolated EVs by SDS-PAGE

The protein concentrations of isolated EVs were determined using Bradford protein assays (Sangon Biotech, Shanghai, China). Proteins (1.5 μg) from isolated *S*. *japonicum* EVs were separated using precast 4–20% polyacrylamide linear gradient gels (Bio-Rad, Hercules, CA, USA). A pre-stained protein standard (Thermo Scientific, Waltham, MA, USA) was used to track protein migration. The gels were sliver-stained as previously described [8] and scanned using a Bio-Rad Molecular Imager FX system (Bio-Rad).

### Enzymatic digestion of protein

The purified EVs samples were dissolved in PBS. To reduce the proteins, dithiothreitol (DTT) was then added at a final concentration of 10 mM followed by incubation at 37°C for 1.5 h. Iodoacetamine (IAA) was then added at a final concentration 50 mM to alkylate the proteins followed by incubation at room temperature in the dark for 40 min. Trypsin was then added at a trypsin to protein ration of 1:50 (w/w) for overnight digestion at 37°C after 4-fold dilution in 25mM NH_4_HCO_3_ buffer to achieve a final urea concentration of less than 2M. Trypsin digestion was stopped by adding trifluoroacetic acid to a final concentration of 1%. The peptides of each sample were desalted on C18 cartridges, concentrated by vacuum centrifugation and reconstituted in 40 μL of 0.1% (v/v) formic acid.

### Mass spectrometry

For total EVs protein identification, LC-MS/MS analysis was performed on a Q Exactive mass spectrometer (Thermo Scientific) that was coupled to an Easy nLC system. Each sample was injected for nanoLC-MS/MS analysis. The peptide mixture was loaded onto a reverse-phase trap column (Thermo Fisher Scientific, Acclaim PepMap100, 100 μm× 2cm, nanoViper C18) connected to a C18-reversed phase analytical column (Thermo Fisher Scientific Easy Column, 10 cm long, 75 μm inner diameter, 3 μm resin) in buffer A (0.1% Formic acid) and separated over a linear gradient of buffer B (84% acetonitrile and 0.1% formic acid) at a flow rate of 300 nL/min controlled by IntelliFlow technology over 60 min. MS data were acquired using a data-dependent top10 method by dynamically choosing the most abundant precursor ions from the survey scan (300–1800 m/z) for high-energy collisional dissociation fragmentation. The automatic gain control target was set to 3e6, and maximum inject time was 10 ms, and dynamic exclusion duration was 40.0 s. Survey scans were acquired at a resolution of 70,000 at m/z 200, and the resolution for the high-energy collisional dissociation spectra was set to 17,500 at m/z 200, with an isolation width of 2 m/z. The normalized collision energy was 30 eV, and the under fill ratio, which specifies the minimum percentage of the target value likely to be reached at the maximum fill time, was defined as 0.1%. The instrument was run with the peptide recognition mode enabled.

### Data analysis

Data interpretation and protein identification were performed with the MS/MS spectra data sets using the Mascot search algorithm (v 2.2, Matrix Science, London, UK) against the UniProtKB *Schistosoma japonicum* database (download on January 22, 2020, with 29,784 entries). The search parameters were trypsin enzyme, two missed cleavages, fixed modifications of carbamidomethyl, variable modifications of oxidation, a fragment ion mass tolerance of 0.10 Da, and peptide tolerance of 20 ppm. Only proteins with at least two peptides (filtered by an ion score ≥20 and false discovery rate of <0.01) uniquely assigned to the respective sequence were considered as identified. Mass spectrometry data along with the identification results have been deposited to the ProteomeXchange Consortium via the PRIDE partner repository with the project accession: PXD018889.

### Gene ontology analysis

To obtain the molecular function, protein class, biological process, and pathway of the proteins involved, we searched the consortium databases of the protein classification software PANTHER (http://www.pantherdb.org/) using *Mus musculus* proteins as a reference.

### Small RNA library preparation and bioinformatics analysis

For the analysis of small RNAs, total RNA was extracted from *S*. *japonicum* EVs using Trizol LS reagent (Thermo Fisher Scientific), and RNA quality was analyzed using an Agilent 2100 system (Agilent Technologies, Santa Clara, CA, USA). Next, the RNA was size-selected using 15% denaturing PAGE; libraries were prepared from the 18–30 nt fraction and ligated first to a 5´RNA adaptor and then to a 3RNA adaptor using an Illumina small RNA Preparation Kit (Illumina, San Diego, CA, USA). The small RNA libraries were subjected to sequencing using Illumina 50 bp single end sequencing performed on an Illumina Hiseq 2000 machine at the BGI (Beijing Genomics Institute, Shenzhen, China). The raw sequencing data were deposited with the NCBI SRA under project number PRJNA547439.

After removing the 3′ adaptor null reads, insert null reads, 5′adaptor contaminants, and reads with a polyA tail, clean datasets were mapped to the draft *S*. *japonicum* genome sequences (sjr2_scaffold.fasta, downloaded from ftp://lifecenter.sgst.cn:2121/nucleotide/corenucleotide) using the Short Oligonucleotide Alignment Program (http://soap.genomics.org.cn). In addition, clean datasets were also analyzed by mapping to the rabbit genome (http://asia.ensembl.org/Oryctolagus_cuniculus/, version 81) to identify host information in the *S*. *japonicum*-secreted EV libraries.

By comparing our sequences with those in databases and selecting the genome location overlap between our data and the databases, sequenced small RNAs were annotated to different categories, including rRNA, tRNA, small nuclear RNA (snRNA), miRNAs, and small nucleolar RNA (snoRNA), by matching against sequences of noncoding RNAs collected in Rfam (Version 11.0) and the NCBI GenBank database (http://www.ncbi.nlm.nih.gov/) using BLAST. For miRNA analysis, these unmatched small RNAs were further analyzed against miRbase (version 21) and GenBank to determine known miRNAs or homologous miRNAs. Finally, unannotated small RNAs were analyzed to identify novel miRNA identification using the Mireap (http://sourceforge.net/projects/mireap).

### RT-qPCR analysis of the abundance of *S*. *japonicum* EV-associated miRNAs and proteins

Total RNAs were isolated from male or female *S*. *japonicum* EVs using TRIzol reagent (Thermo Fisher Scientific) according to the manufacturer’s protocol. The isolated RNA was quantified using a Nanodrop ND-1000 spectrophotometer (Nanodrop Technologies, Wilmington, DE, USA). An miScript II RT Kit (Qiagen, Hilden, Germany) was used to reverse-transcribe the RNA into cDNA. qPCR was performed using an miScript SYBR Green PCR Kit (Qiagen) in a Mastercycler ep realplex (Eppendorf, Hamburg, Germany) as described previously[44]. Briefly, the 20 μL PCR sample contained 2 μL of RT product (1:5 dilution), 10 μL 2×QuantiTect SYBR Green PCR Master Mix, 2 μL 10×miScript Universal Primer, 2 μL 10×miScript Primer Assay and 4 μL H_2_O. The reactions were incubated in a 96-well plate at 95°C for 15 min, followed by 40 cycles of 94°C for 15 s, 55°C for 30 s and 70°C for 30 s. The miScript Primers for *miR-277b*, *miR-277*, *bantam*, *Let-7*, *miR-1*, *miR-71a*, *miR-125b*, *miR-1b*, *miR-61*, *miR-71b and miR-750* were ordered from Qiagen (Qiagen's proprietary). Spiked in cel-miR-39 was used as an internal control for normalizing the abundance of miRNAs associated with EVs between males and females. For determining *miR-750* expression in adult male and female *S*. *japonicum*, qPCR was performed as described above and the *nicotinamide adenine dinucleotide dehydrogenase* gene *(NADH)* was used as an internal control for normalizing the expression of miRNAs between males and females as indicated below.

In addition, adult female schistosomes (28 d) were manually dissected into three parts including anterior, ovary, and vitellarium. The total RNAs were isolated from the pool of each part. RT-qPCR was performed to determine the expression of miR-750 in each part using *NADH* as an internal control.

Total RNAs were isolated from male and female *S*. *japonicum* (28 d) using Trizol (Thermo Fisher Scientific) according to the manufacturer’s instructions. cDNAs were transcribed from total RNA with random primers combined with the oligo dT primer using a PrimeScript RT reagent Kit (TaKaRa, Shiga, Japan), and RT-qPCR analysis was performed using the SYBR Premix ExTaq kit (TaKaRa) according to the manufacturer’s instructions. The detailed primer sequences are shown in S6 Table. The abundance of *NADH* (forward primer: CGA GGA CCT AAC AGC AGA GG; reverse primer: TCC GAA CGA ACT TTG AAT CC) was used as the internal control for normalization. All reactions were performed in triplicate. The 2^−ΔCt^ method was used to calculate the relative miRNA abundance.

### miRNA target prediction

The *miR-750* target gene in *S*. *japonicum* was predicted using miRANDA and TargetScan [45, 46]. Briefly, the source codes of TargetScan (http://www.targetscan.org) and miRANDA 3.3a (http://www.microrna.org) were downloaded and integrated into a home-made software. This software was used *miR-750* sequences and sequences of candidate target regions as input to calculate the base-pairing of a target region with a certain miRNA. Then, the results were filtered by base-pairing type, free energy or context scores to validate that a target region was a miRNA target. The GO analysis of the target genes was performed using BLAST2GO (https://www.blast2go.com).

### Schistosome culture, electroporation, egg counts and morphological observation

*Schistosoma japonicum* were collected from mice at 24–28 days post-infection, and females were separated and cultured in 12-well flat bottom plates containing 2 mL complete RPMI-1640 medium (Thermo Fisher Scientific) supplemented with 2 g/L glucose, 0.3 g/L L-glutamine, 2.0 g/L NaHCO_3_, 15% fetal bovine serum (heat inactivated), and 5% penicillin/streptomycin (10,000 U penicillin and 10 mg/streptomycin in 0.9% NaCl) in a humidified 5% CO_2_ chamber at 37°C. *miR-750* inhibitor (5`AGUUGGAAGCGACAGAUCUGG3`) and scrambled *miR-750* inhibitor (5`AGUUGGCCGCACACCUAUCCG3`) (3 μg per experiment) were modified with 2`-O-methyl (2`-Ome) and Phosphorothioate (PS), chemically synthesized by Shanghai GenePharma (Shanghai, China) were electroporated (125 V, 20 ms, 1 pulse in 200 μL RPMI 1640 medium) into cultured schistosomes. Schistosomes were then transferred into 12-well cell culture plates containing 2 mL fresh media. Eggs were collected from at least three replicate wells containing 30 female worms. The eggs were counted under an inverted microscope (Olympus, Tokyo, Japan) at the indicated times. The parasites were collected at 96 h after electroporation. For microscopic analysis, partial or whole worms were preserved, stained, and mounted and then subjected to morphological examination using confocal microscopy (ZEISS, Oberkochen, Germany), as described previously [29]. Other worm parts were used for RT-qPCR analysis as described below.

### RT-qPCR analysis of the expression of *miR-750* and its target gene transcripts in electroporated schistosomes

At 96 h post electroporation, RNA was isolated from worms using RNAiso plus (Takara) according to the manufacturer’s protocol except for an extended overnight precipitation in isopropanol at -80°C. First strand cDNA was produced from 100 ng RNA using a PrimeScript 1st Strand cDNA Synthesis Kit (Takara) with either a miRNA specific primer (2 μM, 0.5 μL) or random primer combined with Oligo d(T) as described above. qPCR was then carried out to determine the expression of *miR-750* targets using the primers described in S9 Table. *S*. *japonicum NADH* was used as an internal control. Part of the total RNAs were reversely transcribed using a miScript II RT Kit (Qiagen) to determine the expression of *miR-750* using an miScript II RT Kit (Qiagen) as described above. All reactions were performed in at least triplicate. The 2^-ΔCt^ method was used to calculate the relative expression levels.

### Statistical analysis

Results were analyzed using SPSS software (version 17; SPSS, Inc., Chicago, IL, USA). Comparisons between groups were performed using Student’s t tests. Differences were considered significant when *P* ≤ 0.05.

## Supporting information

S1 Table List of proteins identified by MS from EVs isolated from male schistosomes. (XLSX)

S2 Table List of proteins identified by MS from EVs isolated from female schistosomes. (XLSX)

S3 Table List of proteins co-identified from male EVs and female EVs by MS. (XLSX)

S4 Table List of EV marker proteins identified from male and female EVs by MS. (XLSX)

S5 Table List of miRNAs identified from EVs isolated from female and male schistosomes. (XLSX)

S6 Table List of the primers used for qPCR analysis of transcript levels of proteins identified from male and female SjEVs in the study. (XLSX)

S7 Table List of putative targets of miR-750 in *S*. *japonicum*. (XLSX)

S8 Table Result of KEGG analysis of putative targets of *miR-750*. (XLSX)

S9 Table List of the primers used for analyzing the transcript level of *miR-750* targets by qPCR in the study. (XLSX)

S1 Fig Z-Stack of optical sections from *S*. *japonicum* ovary treated with antisense *miR-750*. Female schistosomes treated with *miR-750* inhibitor (antisense *miR-750*) were stained with carmine red and were imaged by confocal microscopy for Z-Stack of optical sections.
(TIF)

S2 Fig Z-Stack of optical sections from *S*. *japonicum* ovary treated with a scrambled antisense *miR-750*. Female schistosomes treated with scrambled *miR-750* inhibitor were stained with carmine red and were imaged by confocal microscopy for Z-Stack of optical sections.
(TIF)
