# Supplementary figures and images for: Proteomic and deep sequencing analysis of extracellular vesicles isolated from adult male and female Schistosoma japonicum
Source: PLoS Negl Trop Dis. 2020 Sep 28;14(9):e0008618. doi: 10.1371/journal.pntd.0008618 (PMC7521736; doi:10.1371/journal.pntd.0008618)

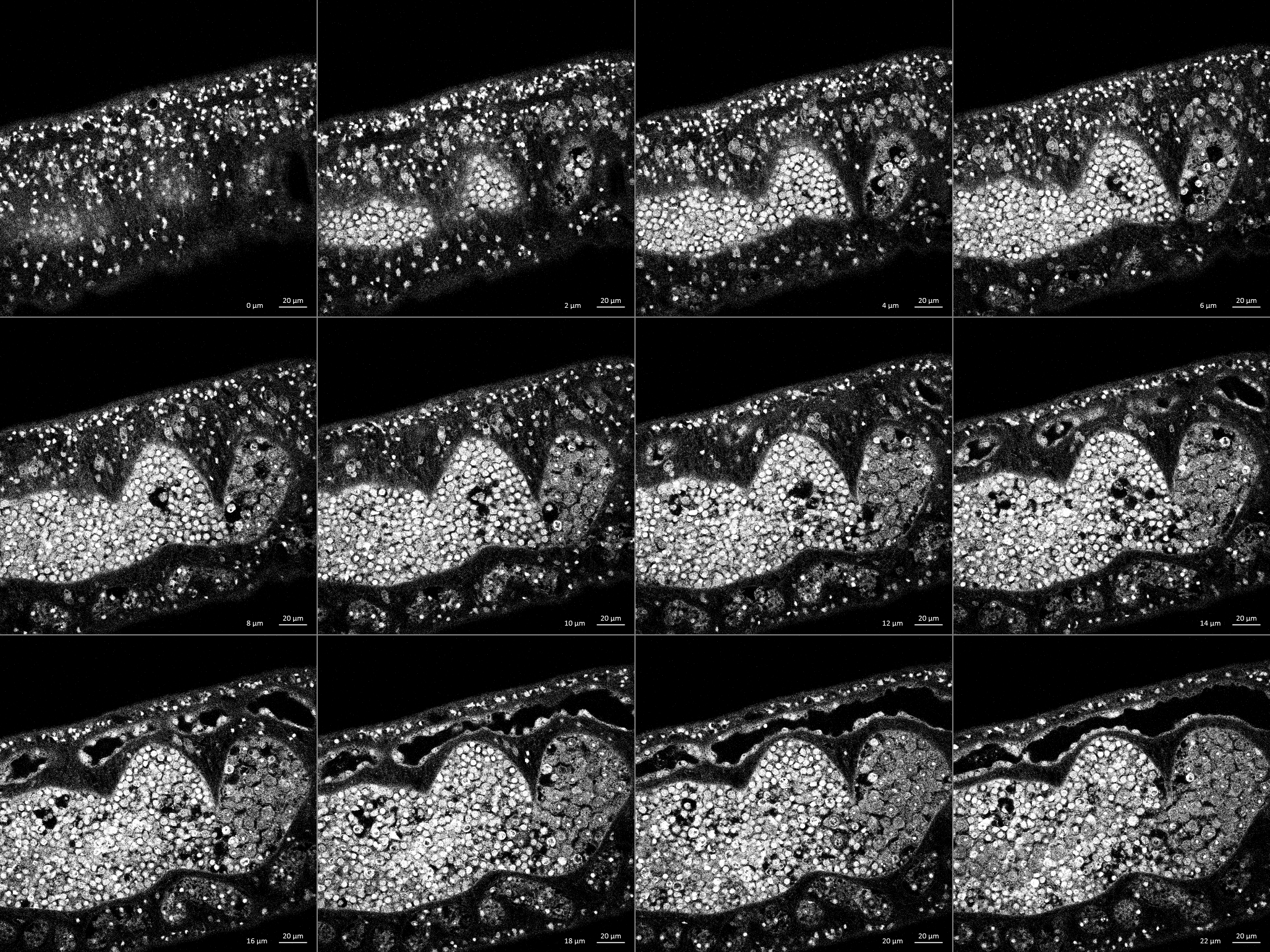

Supplement: S1 Fig — Female schistosomes treated with miR-750 inhibitor (antisense miR-750) were stained with carmine red and were imaged by confocal microscopy for Z-Stack of optical sections. (TIF) [file pntd.0008618.s010.tif]

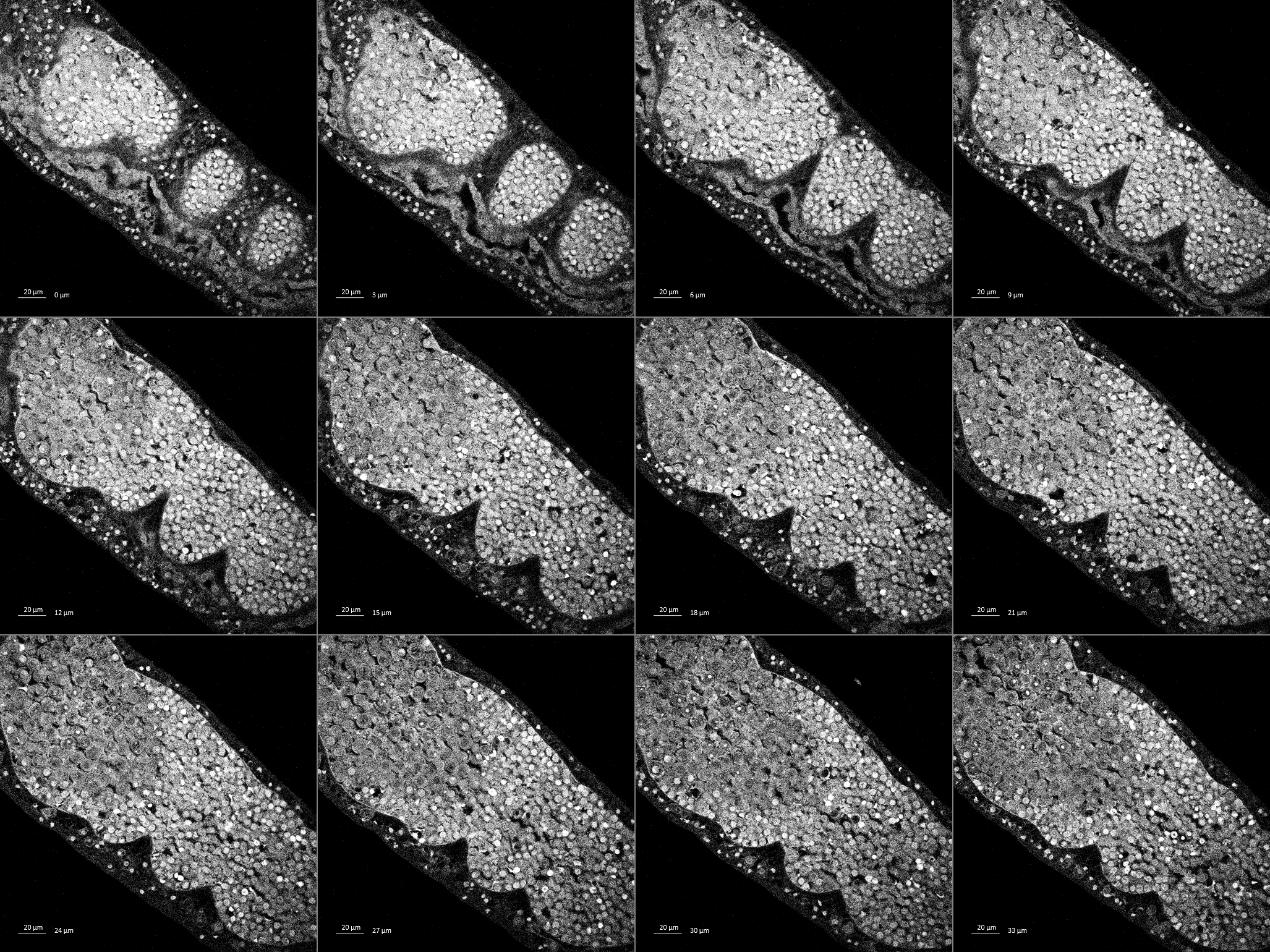

Supplement: S2 Fig — Female schistosomes treated with scrambled miR-750 inhibitor were stained with carmine red and were imaged by confocal microscopy for Z-Stack of optical sections. (TIF) [file pntd.0008618.s011.tif]
